# Toxicity Profiles and Survival Outcomes Among Patients With Nonmetastatic Oropharyngeal Carcinoma Treated With Intensity-Modulated Proton Therapy vs Intensity-Modulated Radiation Therapy

**DOI:** 10.1001/jamanetworkopen.2022.41538

**Published:** 2022-11-11

**Authors:** Irini Youssef, Jennifer Yoon, Nader Mohamed, Kaveh Zakeri, Robert H. Press, Linda Chen, Daphna Y. Gelblum, Sean M. McBride, Chiaojung Jillian Tsai, Nadeem Riaz, Yao Yu, Marc A. Cohen, Lara Ann Dunn, Alan L. Ho, Richard J. Wong, Loren S. Michel, Jay O. Boyle, Bhuvanesh Singh, Anuja Kriplani, Ian Ganly, Eric J. Sherman, David G. Pfister, James Fetten, Nancy Y. Lee

**Affiliations:** 1 Department of Radiation Oncology, SUNY Downstate Health Sciences University, Brooklyn, New York; 2 Department of Radiation Oncology, Rutgers Cancer Institute of New Jersey, New Brunswick; 3 Department of Radiation Oncology, Memorial Sloan Kettering Cancer Center, New York, New York; 4 New York Proton Center, New York; 5 Department of Head and Neck Surgery, Memorial Sloan Kettering Cancer Center, New York, New York; 6 Department of Medical Oncology, Memorial Sloan Kettering Cancer Center, New York, New York

## Abstract

**Importance:**

Patients with oropharyngeal carcinoma (OPC) treated with radiotherapy often experience substantial toxic effects, even with modern techniques such as intensity-modulated radiation therapy (IMRT). Intensity-modulated proton therapy (IMPT) has a potential advantage over IMRT due to reduced dose to the surrounding organs at risk; however, data are scarce given the limited availability and use of IMPT.

**Objective:**

To compare toxic effects and oncologic outcomes among patients with newly diagnosed nonmetastatic OPC treated with IMPT vs IMRT with or without chemotherapy.

**Design, Setting, and Participants:**

This retrospective cohort study included patients aged 18 years or older with newly diagnosed nonmetastatic OPC who received curative-intent radiotherapy with IMPT or IMRT at a single-institution tertiary academic cancer center from January 1, 2018, to December 31, 2021, with follow-up through December 31, 2021.

**Exposures:**

IMPT or IMRT with or without chemotherapy.

**Main Outcomes and Measures:**

The main outcomes were the incidence of acute and chronic (present after ≥6 months) treatment-related adverse events (AEs) and oncologic outcomes, including locoregional recurrence (LRR), progression-free survival (PFS), and overall survival (OS). Fisher exact tests and χ^2^ tests were used to evaluate associations between toxic effects and treatment modality (IMPT vs IMRT), and the Kaplan-Meier method was used to compare LRR, PFS, and OS between the 2 groups.

**Results:**

The study included 292 patients with OPC (272 [93%] with human papillomavirus [HPV]-p16–positive tumors); 254 (87%) were men, 38 (13%) were women, and the median age was 64 years (IQR, 58-71 years). Fifty-eight patients (20%) were treated with IMPT, and 234 (80%) were treated with IMRT. Median follow-up was 26 months (IQR, 17-36 months). Most patients (283 [97%]) received a dose to the primary tumor of 70 Gy. Fifty-seven of the patients treated with IMPT (98%) and 215 of those treated with IMRT (92%) had HPV-p16–positive disease. There were no significant differences in 3-year OS (97% IMPT vs 91% IMRT; *P* = .18), PFS (82% IMPT vs 85% IMRT; *P* = .62), or LRR (5% IMPT vs 4% IMRT; *P* = .59). The incidence of acute toxic effects was significantly higher for IMRT compared with IMPT for oral pain of grade 2 or greater (42 [72%] IMPT vs 217 [93%] IMRT; *P* < .001), xerostomia of grade 2 or greater (12 [21%] IMPT vs 68 [29%] IMRT; *P* < .001), dysgeusia of grade 2 or greater (16 [28%] IMPT vs 134 [57%] IMRT; *P* < .001), grade 3 dysphagia (4 [7%] IMPT vs 29 [12%] IMRT; *P* < .001), mucositis of grade 3 or greater (10 [53%] IMPT vs 13 [70%] IMRT; *P* = .003), nausea of grade 2 or greater (0 [0%] IMPT vs 18 [8%] IMRT; *P* = .04), and weight loss of grade 2 or greater (22 [37%] IMPT vs 138 [59%] IMRT; *P* < .001). There were no significant differences in chronic toxic effects of grade 3 or greater, although there was a significant difference for chronic xerostomia of grade 2 or greater (6 IMPT [11%] vs 22 IMRT [10%]; *P* < .001). Four patients receiving IMRT (2%) vs 0 receiving IMPT had a percutaneous endoscopic gastrostomy tube for longer than 6 months.

**Conclusions and Relevance:**

In this study, curative-intent radiotherapy with IMPT for nonmetastatic OPC was associated with a significantly reduced acute toxicity burden compared with IMRT, with few chronic toxic effects and favorable oncologic outcomes, including locoregional recurrence of only 5% at 2 years. Prospective randomized clinical trials comparing these 2 technologies and of patient-reported outcomes are warranted.

## Key Points

**Question:**

Compared with intensity-modulated radiation therapy (IMRT), is intensity-modulated proton therapy (IMPT) associated with fewer toxic effects and comparable oncologic outcomes among patients with oropharyngeal carcinoma (OPC)?

**Findings:**

In this cohort study of 292 patients with nonmetastatic OPC, IMPT was associated with fewer acute toxic effects compared with IMRT and with few chronic toxic effects. Oncologic outcomes were favorable in both groups, with 5% locoregional recurrence at 2 years with IMPT.

**Meaning:**

The findings suggest that IMPT should be considered as a potential primary radiotherapy modality for nonmetastatic OPC if available because it was associated with less acute toxicity burden and comparable oncologic outcomes vs IMRT.

## Teaser

This cohort study compares the toxic effects and oncologic outcomes associated with intensity-modulated radiation therapy (IMRT) vs intensity-modulated proton therapy (IMPT) among adult patients with oropharyngeal carcinoma.

## Introduction

Oropharyngeal squamous cell carcinoma (OPC) is one of the most common head and neck cancers in the US, annually affecting nearly 20 000 patients.^1^ Despite its rising incidence and prevalence, an increasing proportion of OPC is associated with human papillomavirus (HPV), with a favorable prognosis. Currently, the standard nonsurgical management of nonmetastatic OPC is definitive radiotherapy with or without chemotherapy.^2,3^ Long-term sequelae and quality of life (QOL) are important considerations in the treatment of OPC since patients with OPC are generally young at diagnosis with a potential for long-term survival. Despite the advanced dose conformity provided by the current standard intensity-modulated radiation therapy (IMRT), long-term sequelae^4,5,6,7^ may persist or progress over time and adversely impact QOL in survivors.^8,9,10^

Proton beams have fundamental physical advantages over photon beams, providing a moderate entrance dose, uniform high dose within the tumor, and minimal exit dose with steep distal dose gradients.^11^ These unique dose distributions allow the proton beams to deliver prescription doses to the tumor while sparing nearby normal tissues, whereas the conventional photon beams still deliver a moderate amount of radiation to normal tissues along their path.^12^ The use of intensity-modulated proton therapy (IMPT) in the treatment of OPC has been reported in several studies that showed increased normal-tissue sparing and reduced symptom burden compared with IMRT.^13,14,15^ Despite 2 ongoing phase 3 randomized clinical trials assessing whether IMPT compared with IMRT reduces toxicity in OPC (Toxicity Reduction Using Proton Beam Therapy for Oropharyngeal Cancer [TORPEDO]^16^ and an actively recruiting phase 3 trial at MD Anderson Cancer Center^17^), currently, there are limited phase 3 data. In this study, we examined a cohort of patients with nonmetastatic OPC who were treated with curative-intent IMPT or IMRT to compare the toxicity profiles and survival outcomes.

## Methods

### Patient Cohort

In this cohort study, we retrospectively reviewed all consecutive patients aged 18 years or older with newly diagnosed nonmetastatic OPC who were treated with chemoradiotherapy or radiotherapy (RT) alone at Memorial Sloan Kettering Cancer Center between January 1, 2018, and December 31, 2021. This study was approved by the Memorial Sloan Kettering Cancer Center institutional review board, and a waiver of informed consent was granted because of the retrospective nature of the study. We followed the Strengthening the Reporting of Observational Studies in Epidemiology (STROBE) reporting guideline.

Intensity-modulated proton therapy was generally offered as an alternative to IMRT off trial or was necessitated for large tumors when IMRT could not be safely delivered. The reasons for patients not receiving IMPT included patient preference, insurance denial of IMPT, and logistic issues for making daily visits to the proton centers. The study included patients treated at the Procure Proton Therapy Center, New Jersey, and the New York Proton Center. Proton therapy was delivered using the Proteus 235 Proton Therapy System (Ion Beam Applications) or Varian ProBeam System, treating with either uniform scanning or pencil beam scanning. Pencil beam scanning using 3 to 5 fields with multifield optimization was the preferred modality once available. Setup variations were accounted for by compensator smear or simulated isocenter shift variations of 3 mm and 5 mm, respectively, for both uniform scanning and pencil beam scanning plans. Robustness tests, including a range uncertainty of ±3.5%, were performed to ensure that the clinical target volume receiving at least 95% of the dose was greater than 95%. A generic relative biological effectiveness (RBE) of 1.1 was used for dose evaluation. All patients were aligned using orthogonal radiographs on a 6-df couch.^18^

Patients were immobilized in the treatment position using a 3- or 5-point Aquaplast mask. High-risk clinical target volumes including the gross disease received a dose of 66 to 70 Gy (RBE), while low- to intermediate-risk elective clinical target volumes received a dose of 50 to 54 Gy (RBE). All variations in the primary and neck doses were due to patients missing treatments because of a SARS-CoV-2 infection. At the time of this study, the 30-Gy neck protocol, described elsewhere,^19^ was being adopted as the institutional standard. We used positron emission tomography and computed tomography, computed tomography with contrast if there was no contraindication, and contrast magnetic resonance imaging to delineate the gross primary tumor and nodal volumes treated with a dose of 66 to 70 Gy (RBE). The clinical target volume for the low- to intermediate-risk neck encompassed all regions of potential subclinical disease spread (retropharyngeal, retrostyloid, and levels II to IV for the node-positive neck and levels II to IV for the node-negative neck if receiving RT). The use of simultaneous integrated boost was left to the discretion of the treating physician. Due to resource constraints, we did not routinely do adaptive replanning. However, it is more commonly being used because a benefit has been demonstrated.

Induction or a concurrent chemotherapy regimen was administered at the discretion of the treating medical oncologist. Patients were seen by the radiation oncologist weekly during treatment for toxic effects evaluation. Patients were subsequently followed up at 8 to 12 weeks after the completion of RT, then at 3-month intervals for the first 2 years, and then every 6 to 12 months, with appropriate interval imaging studies and physical examinations.

### Data Collection

Clinicopathologic data were retrospectively curated in a departmental registry. Treatment-related adverse events (AEs) were captured by standardized on-treatment and follow-up notes and verified by 2 of us (I.Y. and J.Y.). Treatment-related AEs were graded using *Common Terminology Criteria for Adverse Events*, version 4.0 (version 5.0 after April 2018). Adverse events occurring within and after 180 days of completion of RT were considered acute and late, respectively. Radiation records and oncologic outcomes were manually abstracted from electronic medical records using a uniform data abstraction form. Locoregional recurrence (LRR) was confirmed by imaging studies or tissue biopsies. Locoregional recurrence, progression-free survival (PFS), and overall survival (OS) were calculated from the start of RT until the occurrence of events or censoring. The end of the follow-up period was December 31, 2021.

### Statistical Analysis

The association of demographic characteristics, clinical characteristics, and treatment factors with treatment modality (IMPT vs IMRT) was assessed using the χ^2^ test or Fisher exact test for categorical covariates and the Wilcoxon rank sum test or Kruskal-Wallis test for continuous variables. The association of acute toxic effects determined a priori and treatment factors with treatment modality were assessed using the χ^2^ test or Fisher exact test. Similarly, the association of chronic toxic effects (present at ≥6 months after treatment) determined a priori and treatment factors with treatment modality were assessed using the χ^2^ test or Fisher exact test.

The association between binary toxicity outcomes and relevant clinical factors were evaluated by univariable logistic regression. Multivariable logistic regression models were then constructed with relevant covariates if the *P* value was less than .20. The Kaplan-Meier method was used to generate and compare LRR, PFS, and OS curves between the IMPT and IMRT groups with a log-rank test. A multivariable Cox proportional hazards regression model was used to calculate hazard ratios (HRs) based on a panel of covariates determined a priori. All tests were 2-sided, and *P* < .05 was considered statistically significant. All analyses were performed using SPSS, version 28.0 (IBM).

## Results

### Patient Characteristics

We identified 292 eligible patients with newly diagnosed nonmetastatic OPC who received curative-intent treatment with RT alone (9 [3%]) or combination systemic therapy and radiation (283 [97%]). Fifty-eight patients (20%) were treated with IMPT, and 234 (80%) were treated with IMRT, with median follow-up of 26 months (IQR, 17-36 months). Median age was 64 years (IQR, 58-71 years); 254 (87%) were men, and 39 (13%) were women. Table 1 summarizes the clinical characteristics by RT modality. Overall, the 2 groups appeared to be balanced in age, sex, smoking history, Karnofsky performance status (KPS), T stage, HPV-p16 status, and use of chemotherapy. The proportion of patients with N0 stage disease was significantly different between the IMPT and IMRT cohorts (10 [17%] vs 17 [7%]), as was the proportion with N1 stage (3 [5%] vs 36 [15%]) and N2C stage (16 [28%] vs 48 [21%]) disease.

**Table 1.** Comparison of Characteristics Between Patients With Oropharyngeal Cancer Receiving IMRT vs IMPT

Characteristic	Patients (N = 292)^a^		*P* value
	IMPT (n = 58)	IMRT (n = 234)	
Age, mean (SD), y	65.2 (9.7)	64.1 (9.1)	.29
Sex			
Female	8 (14)	31 (13)	.52
Male	50 (86)	204 (87)	
Smoking history			
No	34 (59)	117 (50)	.15
Yes	24 (41)	117 (50)	
Smoking duration, mean (SD), pack-years	7.4 (15)	13.2 (20)	.002
Karnofsky performance status^b^			
100	11 (19)	16 (7)	.06
90	40 (69)	169 (72)	
80	7 (12)	47 (20)	
70	0	1 (1)	
60	0	1 (1)	
T stage			
T1	10 (17)	49 (21)	.50
T2	24 (41)	108 (46)	
T3	17 (29)	47 (20)	
T4	7 (12)	30 (13)	
N stage			
N0	10 (17)	17 (7)	.04
N1	3 (5)	36 (15)	
N2A	5 (9)	21 (9)	
N2B	24 (41)	104 (44)	
N2C	16 (28)	48 (21)	
N3	0	8 (3)	
HPV-p16 status			
Positive	57 (98)	215 (92)	.05
Negative or unknown	1 (2)	19 (8)	
Treatment regimen			
Concurrent			.10
Chemotherapy	52 (90)	225 (96)	
Immunotherapy	0	3 (1)	
No systemic therapy	3 (3)	6 (3)	
Type of concurrent chemotherapy			
Cisplatin	50 (86)	186 (80)	.19
Carboplatin/paclitaxel	0	17 (7)	
Cisplatin/paclitaxel	1 (2)	9 (4)	
Carboplatin/5-fluorouracil	1 (2)	7 (3)	
Cisplatin, then switched to alternate therapy due to toxic effects or patient choice	2 (3)	3 (1)	
Other	4 (7)	12 (5)	
Primary dose, median (IQR), GyE	70 (66-78)	70 (64-76)	.11
Neck dose, GyE			
Median (IQR)	3000 (3000-5400)	3000 (3000-5940)	.42
Mean (SD)	3345 (815)	3428 (886)	.18

Abbreviations: GyE, Gray equivalent; HPV, human papillomavirus; IMPT, intensity-modulated proton therapy; IMRT, intensity-modulated radiation therapy.

^a^ Data are presented as number (percentage) of patients unless otherwise indicated.

^b^ Score range 0 to 100, with higher scores indicating higher performance status.

Most patients (236 [81%]) had excellent performance status at the beginning of RT (KPS score, 90-100 [possible score range, 0-100, with higher scores indicating higher functional status]). Most patients (272 [93%]) had HPV-p16–positive disease (57 of those treated with IMPT [98%] vs 215 of those treated with IMRT [92%]). There was no significant difference between the IMPT and IMRT groups in the receipt of concurrent chemotherapy (52 [90%] vs 225 [96%]; *P* = .10) or the receipt of concurrent cisplatin (50 [86%] vs 186 [80%]; *P* = .19). Among patients who were HPV-p16 negative, 11 (53%) received a subclinical neck dose of more than 50 Gy equivalent (GyE), whereas 10 (47%) received doses of 50 GyE or less; in comparison, of the patients who were HPV-p16 positive, 253 (94%) received neck doses of 50 GyE or less (*P* < .001). However, the mean neck dose did not significantly differ between HPV-p16–positive patients and HPV-p16–negative patients.

### Treatment Characteristics and Adverse Events

Most patients (283 [97%]) received biologically equivalent doses (70 GyE) to the primary tumor and grossly involved lymph node. Four patients (1%) received more than 70 GyE, and 2 (1%) received less than 66 GyE. These doses were due to delays in treatments caused by SARS-CoV-2 infection, resulting in missed fractions or requiring additional fractions. Of all patients, 239 (82%) received a subclinical dose of 30 GyE (49 [84%] in the IMPT group vs 190 [81%] in the IMRT group; *P* = .46). One patient in the IMRT group (0.4%) received a neck dose of 59 GyE. The mean and median subclinical doses did not differ significantly between the 2 groups. A diagram of the dose distribution is shown in the eFigure in the Supplement.

Intensity-modulated proton therapy was associated with lower grades of specific acute AEs (Table 2). The incidence of acute toxic effects was significantly higher for IMRT compared with IMPT for oral pain of grade 2 or greater (42 [72%] IMPT vs 217 [93%] IMRT; *P* < .001), xerostomia of grade 2 or greater (12 [21%] IMPT vs 68 [29%] IMRT; *P* < .001), dysgeusia of grade 2 or greater (16 [28%] IMPT vs 134 [57%] IMRT; *P* < .001), grade 3 dysphagia (4 [7%] IMPT vs 29 [12%] IMRT; *P* < .001), mucositis of grade 3 or greater (10 [53%] IMPT vs 13 [70%] IMRT; *P* = .003), nausea of grade 2 or greater (0 [0%] IMPT vs 18 [8%] IMRT; *P* = .04), and weight loss of grade 2 or greater (22 [37%] IMPT vs 138 [59%] IMRT; *P* < .001). Twenty-nine patients in the IMRT group (12%) required percutaneous endoscopic gastrostomy (PEG) tube placement, compared with 4 (7%) in the IMPT group (*P* < .001). Overall, 16 patients receiving IMPT (19%) and 113 receiving IMRT (27%) developed any grade 3 or greater acute AEs (*P* < .001). Additional information on acute toxic effects is reported in Table 2. On univariable analysis, IMPT was the only factor associated with significantly lower likelihood of developing grade 3 or greater acute AEs compared with IMRT (odds ratio [OR], 0.20; 95% CI, 0.02-0.55; *P* = .004) (eTable 1 in the Supplement). No clinicopathological factors were found to be associated with significantly lower likelihood of grade 3 or greater acute AEs. No grade 4 or 5 acute AEs were observed in this study.

**Table 2.** Comparison of Acute Toxic Effects Between Patients With Oropharyngeal Cancer Receiving IMRT vs IMPT

Toxic effect and grade	Patients, No. (%)		*P* value
	IMPT (n = 58)	IMRT (n = 234)	
Oral pain			
0	6 (10)	3 (1)	<.001
1-2	51 (88)	227 (97)	
≥3	1 (2)	3 (2)	
Dermatitis			
0	7 (12)	2 (1)	<.001
1-2	49 (85)	220 (94)	
≥3	2 (3)	12 (5)	
Xerostomia			
0	23 (40)	12 (5)	<.001
1-2	35 (60)	220 (94)	
≥3	0	2 (1)	
Dysgeusia			
0	15 (26)	21 (9)	.002
1-2	43 (74)	213 (91)	
≥3	0	0	
Dysphagia			
0	19 (33)	23 (10)	<.001
1-2	35 (60)	182 (78)	
≥3	4 (7)	29 (12)	
Fatigue			
0	11 (19)	8 (3)	<.001
1-2	47 (81)	222 (95)	
≥3	0	4 (2)	
Mucositis			
0	11 (19)	12 (5)	.003
1-2	41 (71)	192 (82)	
≥3	6 (10)	30 (13)	
Weight loss			
0	9 (15)	0	<.001
1	28 (48)	94 (40)	
2	19 (32)	112 (48)	
≥3	3 (5)	28 (12)	
Hoarseness			
0	51 (88)	157 (67)	<.001
1-2	7 (12)	77 (33)	
≥3	0	0	
Nausea			
0	36 (62)	159 (68)	.40
1-2	22 (38)	70 (30)	
≥3	0	5 (2)	

Abbreviations: IMPT, intensity-modulated proton therapy; IMRT, intensity-modulated radiation therapy.

Records of chronic AEs for 3 patients (5%) in the IMPT group and 12 (5%) in the IMRT group were unavailable because of loss to follow-up after the first post-RT visit. This left 55 patients in the IMPT group and 222 patients in the IMRT group with available data on chronic AEs. Five patients (9%) in the IMPT group and 17 (7%) in the IMRT group developed at least 1 chronic AE of grade 2 or greater, although the difference was not statistically significant (*P* = .56). Only 5 patients (2%) in the IMRT group and no patients in the IMPT group developed at least 1 chronic AE of grade 3 or greater (*P* = .61). Four patients receiving IMRT (2%) and none receiving IMPT had a percutaneous endoscopic gastrostomy tube for 6 months or more after RT. Xerostomia was the only chronic AE for which there was a statistically significant difference in prevalence between IMPT and IMRT (6 patients receiving IMPT [11%] vs 22 receiving IMRT [10%]; *P* *<* .001) (Table 3). Only the use of protons was significantly associated with a lower likelihood of developing any grade 1 or greater chronic AE on univariable analysis (OR, 0.32; 95% CI, 0.14-0.72; *P* = .006) (eTable 2 in the Supplement). No grade 4 or 5 chronic AEs were observed. On multivariable analysis, IMPT was not associated with a significantly lower likelihood of developing any grade 2 or greater chronic AE, compared with IMRT (eTable 2 in the Supplement).

**Table 3.** Comparison of Chronic Toxic Effects Between Patients With Oropharyngeal Cancer Receiving IMRT vs IMPT

Toxic effect and grade	Patients, No. (%)		*P* value
	IMPT (n = 55)	IMRT (n = 222)	
Oral pain			
0	49 (89)	209 (94)	.37
1-2	6 (11)	12 (6)	
≥3	0	1 (1)	
Xerostomia			
0	25 (46)	52 (23)	.001
1-2	30 (55)	170 (77)	
≥3	0	0	
Dysgeusia			
0	37 (67)	131 (59)	.17
1-2	18 (33)	91 (41)	
≥3	0	0	
Dysphagia			
0	47 (86)	190 (86)	.57
1-2	8 (15)	28 (13)	
≥3	0	4 (21)	
Fatigue			
0	47 (85)	197 (89)	.21
1-2	8 (15)	25 (11)	
≥3	0	0	
Hoarseness			
0	55 (100)	220 (99)	.56
1-2	1(0)	2 (1)	
≥3	0	0	
Dermatitis			
0	54 (98)	220 (99)	.56
1-2	1 (2)	2 (1)	
≥3	0	0	
Osteoradionecrosis	1 (2)	10 (5)	.35
Chronic fibrosis^a^	7 (13)	40 (18)	.48
Chronic trismus^a^	2 (4)	14 (6)	.43
Chronic lymphedema^a^	7 (13)	23 (10)	.38

Abbreviations: IMPT, intensity-modulated proton therapy; IMRT, intensity-modulated radiation therapy.

^a^ Present at 6 months or more after treatment.

### Survival Outcomes

The median follow-up times for the entire cohort, IMRT group, and IMPT group were 26 months (IQR, 19-37 months), 26 months (IQR, 19-36 months), and 20 months (IQR, 8-37 months), respectively. Cumulative incidences of LRR in the IMPT vs the IMRT group were estimated to be 5% vs 4%, respectively, at 24 and 36 months (*P* = .59) (Figure, A). On multivariable analysis, treatment modality was not associated with an increased likelihood of LRR, while HPV-p16–positive disease was associated with a lower likelihood of locoregional failure compared with HPV-negative disease (HR, 0.18; 95% CI, 0.05-0.75; *P* = .01). On multivariable Cox proportional hazards regression analyses, HPV status (HR, 0.01; 95% CI, 0.00-0.64) and a higher KPS (HR, 0.72; 95% CI, 0.57-0.93) were associated with decreased risk of LRR.

**Figure.**
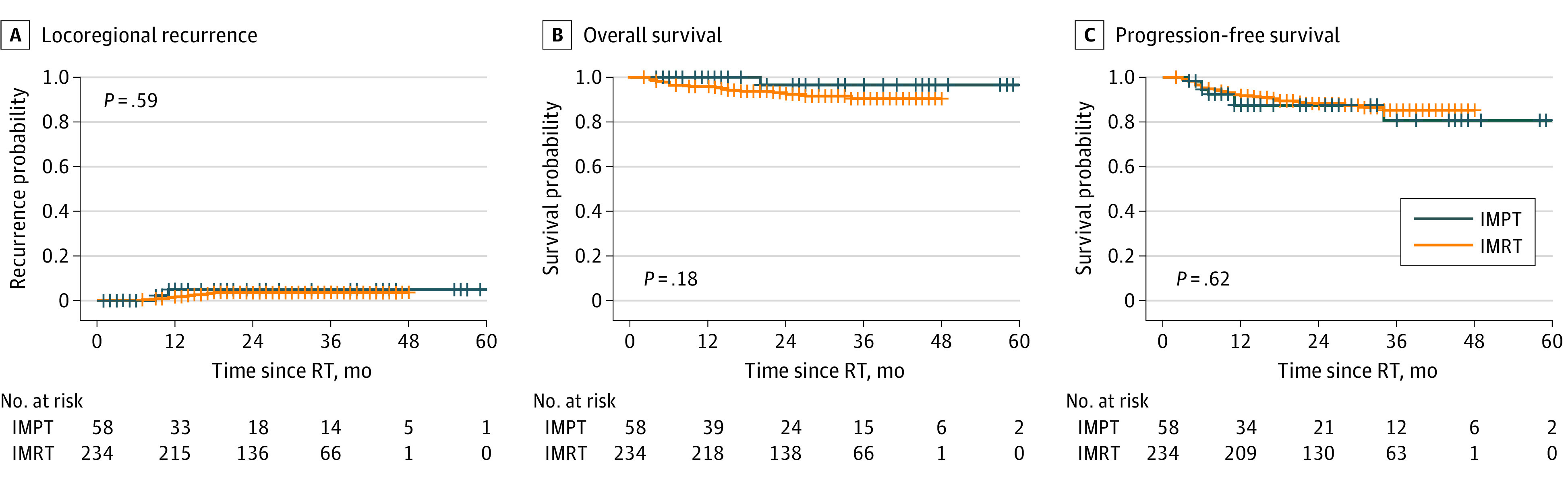
Cumulative Incidence of Overall Survival, Progression-Free Survival, and Locoregional Recurrence by Radiotherapy Modality IMPT indicates intensity-modulated proton therapy; IMRT, intensity-modulated radiation therapy; and RT, radiotherapy.

There was no significant difference in 3-year OS between the IMPT and IMRT groups (97% IMPT vs 91% IMRT; *P* = .18) (Figure, B). On univariable analysis, age, KPS, T stage, HPV status, and use of cisplatin were associated with OS (eTable 3 in the Supplement). On multivariable Cox proportional hazards regression analyses, KPS (HR, 0.92; 95% CI, 0.86-0.98; *P* = .006) and HPV-positive status (HR, 0.14; 95% CI, 0.05-0.38; *P* < .001) were significantly associated with better OS, whereas higher T stage was associated with worse OS (HR, 3.50; 95% CI, 1.19-10.30; *P* = .02). There was no significant difference in PFS between the IMPT and IMRT groups (82% IMPT vs 85% IMPT; *P* = .62) (Figure, C). Performance status, higher N stage, HPV status, and use of cisplatin were associated with better PFS on univariable analysis (eTable 3 in the Supplement). On multivariable analysis, only KPS (HR, 0.90; 95% CI, 0.84-0.96; *P* = .001) and HPV status (HR, 0.23; 95% CI, 0.08-0.62; *P* = .004) remained statistically significant.

## Discussion

The key findings of this study showed that IMPT treatment was significantly associated with reduced treatment-related toxic effects, particularly acute toxic effects. As grade 3 or higher toxic effects usually mean severe symptoms requiring extensive medical intervention with hospitalization and limiting self-care activities of daily living,^20,21^ the reduction of toxic effects and improvement of QOL associated with IMPT treatment could potentially lead to significant savings on health care resources.^22^ It is important to note that most patients in the current cohort received deescalated elective neck doses (30 GyE), suggesting that improvements in acute toxic effects associated with IMPT persisted despite a deescalated strategy with an overall lower expected toxicity burden.^19^

A study by Blanchard et al^14^ found that IMPT was associated with significantly reduced rates of both acute and chronic toxic effects and a trend toward a reduction in the use of PEG tubes. In our study, there was a statistically significant reduction in PEG tube placement associated with IMPT. Of note, the rate of PEG tube placement in the study by Blanchard et al^14^ was as high as 38% in the IMRT group during the acute phase compared with 24% for IMPT, with rates of 23% vs 12%, respectively, 3 months after RT. In contrast, PEG tube placement was lower (≤12%) in both groups in the current study cohort, likely reflecting different practice preferences in supportive care because our institution imposes prompt engagement of a supportive care team early in the treatment course to avoid PEG tube placement unless absolutely necessary or there is failure to thrive due to severe treatment-related toxic effects, such as oral pain, mucositis, and/or dysphagia^22^; a deescalated neck dose of 30 Gy, which became the Memorial Sloan Kettering Cancer Center institutional standard in 2017, was given to 82% of patients in this study.

A study by Manzar et al^15^ compared acute toxic effects and patient-reported outcomes between IMPT and volumetric modulated arc therapy. Overall, IMPT was associated with improved acute toxic effects, including decreased PEG placement, reduced cough and dysgeusia, less hospitalization at 60 or fewer days after RT, and lower narcotic use. These findings suggest that IMPT may be associated with a greater overall absolute reduction in toxic effects for treatments with more expected toxic effects, such as combined modality, 50- to 60-Gy elective neck doses, and full-dose definitive treatments vs postoperative treatments. Improvement in QOL measures has also been shown in several studies. In the study by Manzar et al,^15^ IMPT was associated with improved patient-reported outcomes. A study by Sharma et al^23^ demonstrated higher QOL scores in patients who received pencil beam scanning compared with IMRT; Baumann et al^24^ found reduced hospitalization rates, and Smith et al^25^ reported increased work and productivity recovery trends in patients who received IMPT.

In this study, there was a trend toward a reduction of both acute and chronic grade 3 toxic effects among patients receiving IMPT. After median follow-up close to 2 years for chronic toxic effects associated with IMPT, there were no severe toxic effects. Given that chronic toxic effects among patients with OPC can have an impact on QOL, longer follow-up is necessary. This need will be addressed by the TORPEDO trial^16^ and the actively recruiting phase 3 trial by the MD Anderson Cancer Center.^17^

It is important to note that clinical outcomes were found in this study to be equivalent between IMPT and IMRT, without evidence of in-field or margin misses despite overall improved dose conformality with IMPT. Similar to other studies,^14,26^ our study found comparable cure rates based on both OS and PFS between the IMPT and IMRT groups. While there are ongoing randomized clinical trials aiming to assess whether IMPT can reduce toxic effects in patients with OPC,^16,17^ currently, there are limited available data. Therefore, we believe that our study with a contemporary cohort of patients with nonmetastatic OPC provides valuable data on the toxicity profiles and survival outcomes of IMPT for nonmetastatic OPC. In several studies that reported the outcomes associated with proton therapy,^13,14,15,23,26,27^ the results were based on smaller sample sizes of patients treated with proton therapy, showing the value of our larger study that consisted of 58 patients with nonmetastatic OPC treated with IMPT and 234 treated with IMRT. To our knowledge, this study is the largest retrospective series available at this time that compared IMPT with IMRT for toxicity profiles and survival outcomes in patients with nonmetastatic OPC, which could impact clinical practice. Additional studies comparing proton radiation with photon radiation for oropharyngeal cancer are described in Table 4.

**Table 4.** Studies Investigating Proton Therapy for the Treatment of Oropharyngeal Cancer

Source	Years	Patients, No.	Modality	Stage, No. (%)	Follow-up, mo	Outcome, %	Toxic effects
Sio et al,^13^ 2016	2006-2015	81	IMPT (n = 35); IMRT (n = 46)	IMPT: I, 1 (2.9); II, 1 (2.9); III, 9 (25.7); IVA-B, 24 (68.6); IMRT: I, 1 (2.2); II, 2 (4.4); III, 7 (15.2); IVA-B, 36 (78.3)	IMPT, 7.7; IMRT, 2.68	NA	IMPT: reduced changes in taste and appetite; IMPT had a lower mean MDASI score (5.15) than IMRT (6.58)
Blanchard et al,^14^ 2016	2010-2014	150	IMPT (n = 50); IMRT (n = 100)	T1-T2, 120 (80); T3-T4: 30 (20)	32	IMPT: 3-y OS, 94.3 and 3-y PFS, 86.4; IMRT: 3-y OS, 89.3 and 3-y PFS, 85.8	IMPT: 1-y post-RT PEG rate, 2%, and grade 3 weight loss, 8%; IMRT: 1-y post-RT PEG rate, 7.8%, and grade 3 weight loss, 24.7%
Manzar et al,^15^ 2020	2013-2018	305	IMPT (n = 46); VMAT (n = 259)	Unknown, 4 (1.3); I, 3 (1.0); II, 7 (2.3); III, 23 (7.5); IVA, 245 (80.3); IVB, 17 (5.6); IVC, 6 (2.0)	IMPT, 12; VMAT, 30	NA	IMPT: reduced pain, mucositis, dysphagia, weight loss, and anorexia, but increased mucosal infections and dermatitis
Sharma et al,^23^ 2018	2013-2015	64	PBS (n = 31); VMAT (n = 33)	PBS: I-III, 4 (13); IVA, 27 (87); VMAT: I-III, 5 (15); IVA, 28 (85)	NA	NA	EORTC scale: dental problems at 6-mo follow-up, 17.5 (VMAT) vs 2.0 (PBS); xerostomia at 12-mo follow-up, 54.6 (VMAT) vs 23.5 (PBS); head and neck pain, 22.0 (VMAT) vs 8.3 (PBS)
Yoon et al,^26^ 2021	2016-2019	148	IMRT and IMPT combination (45.3%); IMRT only (54.7%)	IMRT only: I, 0 (0); II, 4 (4.9); III, 11 (13.6); IV, 66 (81.5); IMRT with IMPT: I, 1 (1.5); II, 6 (9.0); III, 10 (14.9); IV, 50 (74.6)	24.7	IMPT: 2-y OS, 100 and 2-y PFS, 89.5; VMAT: 2-y OS, 94.4 and 2-y PFS, 83.7	Acute grade ≥3 toxic effects, IMRT only: dermatitis (3.7%), mucositis (37.0%), weight loss (18.5%), AQA (37.0%); acute grade ≥3 toxic effects, IMRT with IMPT: dermatitis (3.0%), mucositis (13.4%), weight loss (17.9%), AQA (19.4%)
Aljabab et al,^27^ 2020	2015-2017	46	IMPT	I-II, 1 (2); III, 4 (9); IV, 41 (89)	19.2	Last follow-up: LRC, PFS, and OS were 100, 93.5, and 95.7, respectively	Acute grade ≥3 toxic effects: dermatitis (76%), mucositis (72%); no chronic grade ≥4 toxic effects

Abbreviations: AQA, analgesic quantification algorithm; EORTC, European Organisation for Research and Treatment of Cancer; IMPT, intensity-modulated proton therapy; IMRT, intensity-modulated radiation therapy; LRC, locoregional control; MDASI, MD Anderson Symptom Inventory; NA, not applicable; OS, overall survival; PEG, percutaneous endoscopic gastrostomy; PBS, pencil beam scanning; PFS, progression-free survival; RT, radiotherapy; VMAT, volumetric modulated arc therapy.

### Limitations

This study has limitations consistent with its retrospective nature, which limits the strength of conclusions based on potential associations. Another limitation is the small number of patients in the IMPT group, limiting meaningful subset analyses to guide patient selection. However, to our knowledge, this is the only study to date with a large cohort of patients receiving IMPT. Further limitations include the imbalanced median follow-up time between the IMPT and IMRT groups, which was a result of increasingly more patients being treated with IMPT in later years. This could be problematic given that the locoregional failure and chronic complications may occur after 2 years. However, the opposite may also be true as some chronic toxic effects may have already resolved in the IMRT group but have not had time to resolve in the IMPT group given the shorter follow-up times for chronic AEs. In addition, an important confounder, socioeconomic status, was not included in this study, although it may be associated with the affordability of IMPT and is known to impact survival outcomes.^28^ Altogether, however, this large, single-institution comparative study demonstrated that proton beam radiation was associated with few toxic effects and was feasible for the treatment of patients with oropharyngeal cancer, without compromising oncologic outcomes.

## Conclusions

In this cohort study, primary IMPT for nonmetastatic OPC was significantly associated with a reduced acute toxicity burden compared with IMRT, with few severe chronic adverse effects and favorable oncologic outcomes. In particular, PEG tube placement was significantly reduced in patients receiving IMPT during the acute phase and absent during the chronic phase. The findings suggest that IMPT is well tolerated in the setting of nonmetastatic, de novo OPC. Prospective randomized clinical trials along with patient-reported outcomes are warranted to optimize patient selection for IMPT in nonmetastatic OPC, especially given that many of these patients have a potential for long-term survival.

## Notes

Supplement. **eFigure.** Percentage of Patients Receiving Dose by Treatment Modality **eTable 1.** Univariable and Multivariable Analyses for Factors Associated With the Development of Grade 3 or Higher Acute Adverse Events **eTable 2.** Univariable and Multivariable Analyses for Factors Associated With the Development of Grade 1 or Higher Chronic Adverse Events **eTable 3.** Univariable and Multivariable Analyses for Overall Survival and Progression-Free Survival

## References

[1] Damgacioglu H, Sonawane K, Zhu Y, . Oropharyngeal cancer incidence and mortality trends in all 50 states in the US, 2001-2017. JAMA Otolaryngol Head Neck Surg. 2022;148(2):155-165. doi:10.1001/jamaoto.2021.3567 34913945 PMC8678903

[2] Gillison ML, Chaturvedi AK, Anderson WF, Fakhry C. Epidemiology of human papillomavirus-positive head and neck squamous cell carcinoma. J Clin Oncol. 2015;33(29):3235-3242. doi:10.1200/JCO.2015.61.6995 26351338 PMC4979086

[3] Dillon MT, Harrington KJ. Human papillomavirus-negative pharyngeal cancer. J Clin Oncol. 2015;33(29):3251-3261. doi:10.1200/JCO.2015.60.7804 26351347

[4] Chera BS, Amdur RJ, Tepper J, . Phase 2 trial of de-intensified chemoradiation therapy for favorable-risk human papillomavirus-associated oropharyngeal squamous cell carcinoma. Int J Radiat Oncol Biol Phys. 2015;93(5):976-985. doi:10.1016/j.ijrobp.2015.08.033 26581135

[5] Garden AS, Dong L, Morrison WH, . Patterns of disease recurrence following treatment of oropharyngeal cancer with intensity modulated radiation therapy. Int J Radiat Oncol Biol Phys. 2013;85(4):941-947. doi:10.1016/j.ijrobp.2012.08.004 22975604

[6] Gillison ML, Trotti AM, Harris J, . Radiotherapy plus cetuximab or cisplatin in human papillomavirus-positive oropharyngeal cancer (NRG Oncology RTOG 1016): a randomised, multicentre, non-inferiority trial. Lancet. 2019;393(10166):40-50. doi:10.1016/S0140-6736(18)32779-X 30449625 PMC6541928

[7] Mallick I, Waldron JN. Radiation therapy for head and neck cancers. Semin Oncol Nurs. 2009;25(3):193-202. doi:10.1016/j.soncn.2009.05.002 19635398

[8] Barnett GC, West CM, Dunning AM, . Normal tissue reactions to radiotherapy: towards tailoring treatment dose by genotype. Nat Rev Cancer. 2009;9(2):134-142. doi:10.1038/nrc2587 19148183 PMC2670578

[9] Jellema AP, Slotman BJ, Doornaert P, Leemans CR, Langendijk JA. Impact of radiation-induced xerostomia on quality of life after primary radiotherapy among patients with head and neck cancer. Int J Radiat Oncol Biol Phys. 2007;69(3):751-760. doi:10.1016/j.ijrobp.2007.04.021 17560735

[10] Langendijk JA, Doornaert P, Verdonck-de Leeuw IM, Leemans CR, Aaronson NK, Slotman BJ. Impact of late treatment-related toxicity on quality of life among patients with head and neck cancer treated with radiotherapy. J Clin Oncol. 2008;26(22):3770-3776. doi:10.1200/JCO.2007.14.6647 18669465

[11] Miller DW. A review of proton beam radiation therapy. Med Phys. 1995;22(11 Pt 2):1943-1954. doi:10.1118/1.597435 8587548

[12] Leeman JE, Romesser PB, Zhou Y, . Proton therapy for head and neck cancer: expanding the therapeutic window. Lancet Oncol. 2017;18(5):e254-e265. doi:10.1016/S1470-2045(17)30179-1 28456587

[13] Sio TT, Lin HK, Shi Q, . Intensity modulated proton therapy versus intensity modulated photon radiation therapy for oropharyngeal cancer: first comparative results of patient-reported outcomes. Int J Radiat Oncol Biol Phys. 2016;95(4):1107-1114. doi:10.1016/j.ijrobp.2016.02.044 27354125 PMC5409532

[14] Blanchard P, Garden AS, Gunn GB, . Intensity-modulated proton beam therapy (IMPT) versus intensity-modulated photon therapy (IMRT) for patients with oropharynx cancer—a case matched analysis. Radiother Oncol. 2016;120(1):48-55. doi:10.1016/j.radonc.2016.05.022 27342249 PMC5474304

[15] Manzar GS, Lester SC, Routman DM, . Comparative analysis of acute toxicities and patient reported outcomes between intensity-modulated proton therapy (IMPT) and volumetric modulated arc therapy (VMAT) for the treatment of oropharyngeal cancer. Radiother Oncol. 2020;147:64-74. doi:10.1016/j.radonc.2020.03.010 32234612

[16] Price J, Hall E, West C, Thomson D. TORPEdO—a phase III trial of intensity-modulated proton beam therapy versus intensity-modulated radiotherapy for multi-toxicity reduction in oropharyngeal cancer. Clin Oncol (R Coll Radiol). 2020;32(2):84-88. doi:10.1016/j.clon.2019.09.052 31604604

[17] Intensity-modulated proton beam therapy or intensity-modulated photon therapy in treating patients with stage III-IVB oropharyngeal cancer. ClinicalTrials.gov identifier: NCT01893307. Accessed May 25, 2022. https://clinicaltrials.gov/ct2/show/NCT01893307

[18] Zakeri K, Wang H, Kang JJ, . Outcomes and prognostic factors of major salivary gland tumors treated with proton beam radiation therapy. Head Neck. 2021;43(4):1056-1062. doi:10.1002/hed.26563 33606323 PMC9371938

[19] Tsai CJ, McBride SM, Riaz N, . Evaluation of substantial reduction in elective radiotherapy dose and field in patients with human papillomavirus-associated oropharyngeal carcinoma treated with definitive chemoradiotherapy. JAMA Oncol. 2022;8(3):364-372. doi:10.1001/jamaoncol.2021.6416 35050342 PMC8778604

[20] Cancer Therapy Evaluation Program. Common Terminology Criteria for Adverse Events (CTCAE) Version 5.0. National Cancer Institute, National Institutes of Health. Published November 27, 2017. Accessed January 12, 2021. https://ctep.cancer.gov/protocoldevelopment/electronic_applications/docs/ctcae_v5_quick_reference_5x7.pdf

[21] Al-Mamgani A, Van Rooij P, Tans L, Teguh DN, Levendag PC. Toxicity and outcome of intensity-modulated radiotherapy versus 3-dimensional conformal radiotherapy for oropharyngeal cancer: a matched-pair analysis. Technol Cancer Res Treat. 2013;12(2):123-130. doi:10.7785/tcrt.2012.500305 23098281

[22] Li X, Kitpanit S, Lee A, . Toxicity profiles and survival outcomes among patients with nonmetastatic nasopharyngeal carcinoma treated with intensity-modulated proton therapy vs intensity-modulated radiation therapy. JAMA Netw Open. 2021;4(6):e2113205. doi:10.1001/jamanetworkopen.2021.13205 34143193 PMC8214161

[23] Sharma S, Zhou O, Thompson R, . Quality of life of postoperative photon versus proton radiation therapy for oropharynx cancer. Int J Part Ther. 2018;5(2):11-17. doi:10.14338/IJPT-18-00032.1 31773030 PMC6874189

[24] Baumann BC, Mitra N, Harton JG, . Comparative effectiveness of proton vs photon therapy as part of concurrent chemoradiotherapy for locally advanced cancer. JAMA Oncol. 2020;6(2):237-246. doi:10.1001/jamaoncol.2019.4889 31876914 PMC6990870

[25] Smith GL, Fu S, Ning MS, . Work outcomes after intensity-modulated proton therapy (IMPT) versus intensity-modulated photon therapy (IMRT) for oropharyngeal cancer. Int J Part Ther. 2021;8(1):319-327. doi:10.14338/IJPT-20-00067.1 34285958 PMC8270077

[26] Yoon HG, Ahn YC, Oh D, . Early clinical outcomes of intensity modulated radiation therapy/intensity modulated proton therapy combination in comparison with intensity modulated radiation therapy alone in oropharynx cancer patients. Cancers (Basel). 2021;13(7):1549. doi:10.3390/cancers13071549 33801766 PMC8037748

[27] Aljabab S, Liu A, Wong T, Liao JJ, Laramore GE, Parvathaneni U. Proton therapy for locally advanced oropharyngeal cancer: initial clinical experience at the University of Washington. Int J Part Ther. 2020;6(3):1-12. 32582809 10.14338/IJPT-19-00053.1 PMC7038913

[28] Cella DF, Orav EJ, Kornblith AB, . Socioeconomic status and cancer survival. J Clin Oncol. 1991;9(8):1500-1509. doi:10.1200/JCO.1991.9.8.1500 2072149

