# Errors in Abstract and Results and Addition of Corresponding Author and Acknowledgments

**DOI:** 10.1001/jamanetworkopen.2022.50485

**Published:** 2022-12-20

In the Original Investigation titled “Toxicity Profiles and Survival Outcomes Among Patients With Nonmetastatic Oropharyngeal Carcinoma Treated With Intensity-Modulated Proton Therapy vs Intensity-Modulated Radiation Therapy,”^1^ published November 11, 2022, in the abstract Results, “There were no significant differences in chronic toxic effects, although there was a significant difference for chronic xerostomia of grade 2 or greater (6 IMPT [11%] vs 22 IMRT [10%]; *P* < .001)” was changed to “There were no significant differences in chronic toxic effects except for xerostomia.” In the Treatment Characteristics and Adverse Events subsection of the Results section, “Xerostomia was the only chronic AE for which there was a statistically significant difference in prevalence between IMPT and IMRT (6 patients receiving IMPT [11%] vs 22 receiving IMRT [10%]; *P* *<* .001)” was changed to “Xerostomia was the only chronic AE for which there was a statistically significant difference in toxicity between IMPT and IMRT.” In addition, Nancy Y. Lee, MD, was added as a corresponding author, and an Acknowledgments section was added. This article has been corrected.^1^
