# Supplementary material for: Toxicity Profiles and Survival Outcomes Among Patients With Nonmetastatic Oropharyngeal Carcinoma Treated With Intensity-Modulated Proton Therapy vs Intensity-Modulated Radiation Therapy
Source: JAMA Netw Open. 2022 Nov 11;5(11):e2241538. doi: 10.1001/jamanetworkopen.2022.41538 (PMC9652753; doi:10.1001/jamanetworkopen.2022.41538)
Supplement: Supplement. — eFigure. Percentage of Patients Receiving Dose by Treatment Modality eTable 1. Univariable and Multivariable Analyses for Factors Associated With the Development of Grade 3 or Higher Acute Adverse Events eTable 2. Univariable and Multivariable Analyses for Factors Associated With the Development of Grade 1 or Higher Chronic Adverse Events eTable 3. Univariable and Multivariable Analyses for Overall Survival and Progression-Free Survival [file jamanetwopen-e2241538-s001.pdf]

Supplementary Online Content

Youssef I, Yoon J, Mohamed N, et al. Toxicity profiles and survival outcomes among patients with nonmetastatic oropharyngeal carcinoma treated with intensity-modulated proton therapy vs intensity-modulated radiation therapy. *JAMA Netw Open*. 2022;5(11):e2241538. doi:10.1001/jamanetworkopen.2022.41538

eFigure. Percentage of Patients Receiving Dose by Treatment Modality
eTable 1. Univariable and Multivariable Analyses for Factors Associated With the Development of Grade 3 or Higher Acute Adverse Events
eTable 2. Univariable and Multivariable Analyses for Factors Associated With the Development of Grade 1 or Higher Chronic Adverse Events
eTable 3. Univariable and Multivariable Analyses for Overall Survival and Progression-Free Survival

This supplementary material has been provided by the authors to give readers additional information about their work.

© 2022 Youssef I et al. *JAMA Network Open*.

eFigure

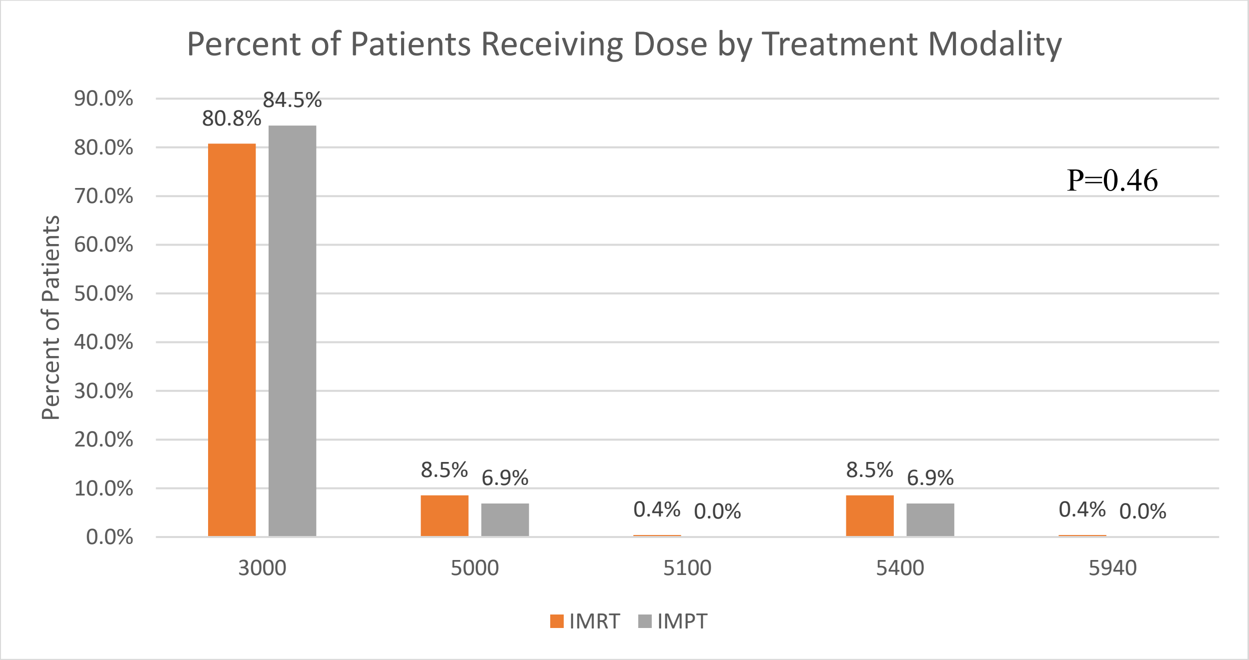

© 2022 Youssef I et al. *JAMA Network Open*.

eTable 1: Univariable and Multivariable Analyses for Factors Associated With the Development of Grade 3 or Higher Acute Adverse Events				
Variable	UVA [Confidence Interval-(CI)]	P value	MVA (Confidence Interval)	P value
RT Modality				
IMRT	1 [Reference]			
IMPT	0.20 [0.015-0.55]	**.004**		
Sex				
Female	1 [Reference]			
Male	1.66 [0.34-8.1]	0.534		
Age	0.96 [0.89-1.03]	0.214		
Smoking History				
No	1 [Reference]			
Yes	1.42 [0.39-5.1]	0.595		
KPS	.998 [0.89-1.12]	0.977		
T-Stage				
T1-T2	1 [Reference]			
T3-T4	0.799 [0.22-2.9]	0.733		
N stage				
N0-N1	1 [Reference]			
N2-N3	1.92 [0.42-8.82]	0.40		
HPV/P16 status				
Negative/UK	1 [Reference]			
Positive	---- [a]	NA		
Concurrent systemic therapy				
Yes	1 [Reference]			
No	---- [a]	NA		
Type of Chemotherapy				
Cisplatin	1 [Reference]			
Other	0.76 [0.16-3.69]	0.73		

Abbreviations: UVA: Univariable Analysis, MVA: Multivariable analysis, IMRT: Intensity Modulated Radiation Therapy, IMPT: Intensity Modulated Proton Therapy, HPV, Human Papilloma Virus

a-OR and CI could not be computed for HPV/p16 status and Concurrent systemic therapy due to zero values

*MVA cells empty since only 1 variable was significant on UVA

© 2022 Youssef I et al. *JAMA Network Open*.

eTable 2: Univariable and Multivariable Analyses for Factors Associated With the Development of Grade 1 or Higher Chronic Adverse Events				
Variable	UVA [Confidence Interval-(CI)]	P value	MVA* (Confidence Interval)	P value
RT Modality				
IMRT	1 [Reference]			
IMPT	0.32 [0.14-0.72]	**.006**		
Sex				
Female	1 [Reference]			
Male	0.55 [0.20-1.47]	0.23		
Age	1.01 [0.96-1.05]	0.80		
Smoking History				
No	1 [Reference]			
Yes	1.15 [0.54-2.5]	0.72		
Karnofsky Performance Status	1.02 [0.95-1.09]	0.63		
T-Stage				
T1-T2	1 [Reference]			
T3-T4	0.82 [0.37-1.8]	0.63		
N stage				
N0-N1	1 [Reference]			
N2-N3	1.45 [0.54-3.9]	0.46		
HPV/P16 status				
Negative/Unknown	1 [Reference]			
Positive	1.79 [0.48-6.6]	0.39		
Concurrent systemic therapy				
Yes	1 [Reference]			
No	0.88 [.11-7.21]	0.91		
Type of Chemotherapy				
Cisplatin	1 [Reference]			
Other	1.6[0.46-5.54]	0.46		

Abbreviations: UVA: Univariable Analysis, MVA: Multivariable analysis, IMRT: Intensity Modulated Radiation Therapy, IMPT: Intensity Modulated Proton Therapy, HPV, Human Papilloma Virus
*MVA cells empty since only 1 variable was significant on UVA

© 2022 Youssef I et al. *JAMA Network Open*.

eTable 3: Univariable and Multivariable Analyses for Overall Survival and Progression-Free Survival								
	Overall Survival				Progression Free Survival			
Variable	Univariable		Multivariable		Univariable		Multivariable	
	HR[CI]	P value	HR	P value	HR	P value	HR	P value
Radiation Modality								
IMRT	1 [Reference]		1 [Reference]		1 [Reference]		1 [Reference]	
IMPT	0.28 [.04-2.1]	0.21			1.23[.54-2.82]	0.62	NA	NA
Sex								
Male	1 [Reference]				1 [Reference]		1 [Reference]	
Female	1.25[.36-4.28]	0.73			.83 [.29-2.34]	.72		
Age	1.04[0.99-1.1]	**0.17**	1.0 [.96-1.07]	.60	1.04 [1-1.08]	**.06**	1.05[.99-1.1]	**.07**
Smoking History								
No	1 [Reference]				1 [Reference]		1 [Reference]	
Yes	1.76[0.69-4.5]	0.226			1.68[0.89-3.28]	0.163		
Karnofsky Performance Status	0.9[.86-.95]	**<.001**	.92 [.86-.98]	**.006**	.92 [0.88-0.96]	**<.001**	0.9 [.84-.96]	**.001**
T Stage								
T1-T2	1 [Reference]		1 [Reference]		1 [Reference]		1 [Reference]	
T3-T4	5.77 [2.1-16.03]	**<.001**	3.5 [1.19-10.3]	**.02**	3.98[1.99-7.97]	**<.001**	1.84 [.74-4.56]	.19
N stage								
N0-N1	1 [Reference]				1 [Reference]		1 [Reference]	
N2-N3	.66 [.192-2.25]	.50			.59[0.27-1.32]	**0.20**	.85 [.34-2.12]	0.85
HPV/P16 status								
Negative/Unknown	1 [Reference]		1 [Reference]		1 [Reference]		1 [Reference]	
Positive	.15 [.06-.39]	**<.001**	.14 [.05-.38]	**<.001**	0.27 [.12-.62]	**.002**	.23 [.08-.62]	**.004**
Concurrent systemic therapy								
No	1 [Reference]				1 [Reference]		1 [Reference]	
Yes	21.2[0-12]	.56			21.2[.01-65]	0.46		
Type of Chemotherapy								
Other	1 [Reference]		1 [Reference]		1 [Reference]		1 [Reference]	
Cisplatin	4.05 [1.63-10.1]	**.003**	2.05 [.76-5.55]	.158	2.77[1.38-5.50]	**.004**	1.31 [0.5-3.5]	0.6

Abbreviations: UVA: Univariable Analysis, MVA: Multivariable analysis, IMRT: Intensity Modulated Radiation Therapy, IMPT: Intensity Modulated Proton Therapy, HPV, Human Papilloma Virus

© 2022 Youssef I et al. *JAMA Network Open*.
